# Serious physical fighting and gambling-related attitudes and behaviors in adolescents

**DOI:** 10.1556/JBA.2.2013.009

**Published:** 2013-06-14

**Authors:** Melissa Slavin, Corey E. Pilver, Rani A. Hoff, Suchitra Krishnan-Sarin, Marvin A. Steinberg, Loreen Rugle, Marc N. Potenza

**Affiliations:** ^1^Child Study Center, Yale University School of Medicine, New Haven, CT, USA; ^2^School of Public Health, Yale University, New Haven, CT, USA; ^3^National Center for PTSD, Evaluation Division, VA CT Healthcare System, West Haven, CT, USA; ^4^Department of Psychiatry, Yale University School of Medicine, Connecticut Mental Health Center, New Haven, CT, USA; ^5^The Connecticut Council on Problem Gambling, Clinton, CT, USA; ^6^Connecticut Problem Gambling Services, Middletown, CT, USA; ^7^Department of Neurobiology, Yale University School of Medicine, New Haven, CT, USA

**Keywords:** fighting, gambling, physical violence, adolescents, risk behaviors, high school

## Abstract

*Background and aims:* Physical fighting and gambling are common risk behaviors among adolescents. Prior studies have found associations among these behaviors in adolescents but have not examined systematically the health and gambling correlates of problem-gambling severity amongst youth stratified by fight involvement. *Methods:* Survey data were used from 2,276 Connecticut high school adolescents regarding their physical fight involvement, gambling behaviors and perceptions, and health and functioning. Gambling perceptions and correlates of problem-gambling severity were examined in fighting and non-fighting adolescents. *Results:* Gambling perceptions were more permissive and at-risk/problem gambling was more frequent amongst adolescents reporting serious fights versus those denying serious fights. A stronger relationship between problem-gambling severity and regular smoking was observed for adolescents involved in fights. *Discussion and conclusions:* The more permissive gambling attitudes and heavier gambling associated with serious fights in high school students suggest that youth who engage in physical fights warrant enhanced prevention efforts related to gambling. The stronger relationship between tobacco smoking and problem-gambling severity amongst youth engaging in serious fights suggest that fighting youth who smoke might warrant particular screening for gambling problems and subsequent interventions.

## Introduction

Both physical fighting and gambling are risk behaviors that occur often among adolescents and each represents a significant health concern. A recent study found that 33% of high school students reported involvement in physical fights in 2011, with 12% of such fights occurring on school property (Eaton et al., 2012). Fighting is associated with other high-risk behaviors including early sex, substance use, and lower academic achievement (Dukarm, Byrd, Auinger & Weitzman, 1996; Fraga, Ramos, Dias & Barros, 2011; Howard, Wang & Yan, 2007, 2008; Pickett et al., 2005). Estimates of past-year gambling among adolescents are even higher, ranging from 50–90% (Gupta & Derevensky, 2000; Shaffer & Hall, 2001). Gambling, particularly at-risk or problem gambling (ARPG) which reflects greater problem-gambling severity, has been associated with poor academic functioning, violence, depression and substance abuse, and problems later in life (Potenza et al., 2011; Rahman et al., 2012). While parents and adolescents appear aware of problems associated with fighting (Hamburg, 1998; St. George & Thomas, 1997), data suggest that both groups may be less concerned about the risks associated with adolescent gambling (Campbell, Derevensky, Meerkamper & Cutajar, 2012).

Gambling and aggression, particularly extreme patterns of each (e.g., ARPG and propensities to get into physical fights) might each be conceptualized as expressions of impaired behavioral impulse control and thus be hypothesized to be associated. Gambling and fighting have been shown to co-occur amongst adults (Afifi, Brownridge, MacMillan & Sareen, 2010; Brasfield et al., 2012; Korman et al., 2008) and adolescents (Chaumeton, Ramowski & Nystrom, 2011; Goldstein, Walton, Cunningham, Resko & Duan, 2009; Potenza et al., 2011; Proimos, DuRant, Pierce & Goodman, 1998), suggesting that adolescents who fight might view gambling more permissively, and vice versa. However, little is known regarding their interaction and relative impacts on adolescent health. Despite associations between gambling and fighting, prior studies have not systematically examined health and gambling correlates of problem-gambling severity amongst adolescents based on their involvement in serious physical fights.

To address an existing gap in knowledge, we examined high school survey data to investigate the relationship between problem-gambling severity and health and gambling measures in adolescents who acknowledged or denied past-year involvement in serious fights resulting in physical injury. This subsample of adolescents, which may include victims of bullying, perpetrators of bullying, or individuals involved in both bullying and victimization, is a population associated with a variety of aggressive and risky behaviors. A number of studies have shown that victims of bullying, as well as adolescents defined as both bullies and victims, have reported a greater level of substance use than adolescents uninvolved in bullying behaviors (Radliff, Wheaton, Robinson & Morris, 2012; Tharp-Taylor, Haviland & D’Amico, 2009). Links between risky behaviors and victimization have also been observed in adults, with over 50% of one sample of problem gamblers reporting past subjection to physical and verbal intimate partner abuse (Korman et al., 2008). Often it is challenging to ascertain through self-report the extent to which adolescents involved in physical aggression may be perpetrators, victims or both. For these reasons, the group as being involved in physical fights was considered as a single entity.

In this current study, we hypothesized that problem-gambling severity would be associated with fight involvement; adolescents involved in fights would view gambling more permissively and problem-gambling prevention efforts as less important; and health and functioning measures (poor academic performance, carrying a weapon, and substance use) and gambling measures (types and locations of gambling) would show differential relationships with problem-gambling severity in the fighting versus non-fighting groups (e.g., given propensities to fight on school grounds, different relationships with gambling on school grounds would be observed).

## Methods

### Survey

Cross-sectional, anonymous survey data from high school students were collected as described previously (Cavallo et al., 2010; Desai, Krishnan-Sarin, Cavallo & Potenza, 2010; Grant, Potenza, Krishnan-Sarin, Cavallo & Desai, 2011a, 2011b; Kundu et al., in press; Liu, Desai, Krishnan-Sarin, Cavallo & Potenza, 2011; Potenza et al., 2011; Rahman et al., 2012; Schepis et al., 2008, 2010; Yip et al., 2011). Every public 4-year and non-vocational and special-education high school in Connecticut was invited to participate. The initial response from schools was not sufficient to ensure representation of all geographic regions in Connecticut so schools in targeted areas were re-contacted. The final sample included schools from each geographic quadrant and all three district-reference groups (a proximal link to socioeconomic status) and was consistent with the 2000 Census data of 14- to 18-year-old Connecticut residents. For the current study, 2,276 adolescents who completed the serious-fighting measure and all 12 questions corresponding to the inclusionary criteria for pathological gambling were included. A passive-consent procedure was utilized to obtain parental permission. Letters were mailed to parents outlining the study and instructing those not wanting their child participating to contact their child's high school, usually by calling the school's main office. From these phone calls, a list of students who were ineligible to participate was compiled for use on survey administration day. This consent procedure was approved by participating schools and Yale's Institutional Review Board.

Survey administration occurred on a single day at each school. Participation was voluntary, taking around 50 minutes. Reminders were given to keep information anonymous. Less than 1% of students refused to participate.

### Measures

*Problem-gambling severity and fight measures.* Problem-gambling severity was defined (non-gambling, low-risk gambling [LRG], at-risk/problem gambling [ARPG]) using the 12 items from the Massachusetts Gambling Screen (MAGS) relating to the 10 inclusionary criteria for DSM-IV pathological gambling (Potenza et al., 2011; Shaffer, LaBrie, Scanlan & Cummings, 1994; Yip et al., 2011). The MAGS is a validated instrument designed to assess DSM-IV pathological gambling in adolescents (Shaffer et al., 1994). Specifically, participants endorsing gambling and no inclusionary criteria were classified as having LRG and those endorsing one or more criteria were classified as having ARPG.

Respondents were categorized into fight and non-fight groups based on a question from the Youth Behavior Risk Survey that stated, “During the past 12 months, how many times were you in a physical fight in which you were injured and had to be treated by a doctor or nurse?”, with responses grouped as one or more versus none (Eaton et al., 2012).

*Gambling perceptions.* As described previously (Kundu et al., in press), adolescents indicated the importance (very/somewhat = “important” versus “not important”) of the following gambling prevention approaches: checking identification for purchasing lottery tickets; hanging out with friends who do not gamble; participating in activities that are fun and free of gambling; fear of losing valuable possessions, friends or relatives to gambling; advertisements that show problems associated with gambling; not having access to Internet gambling at home; parent/guardian strictness about gambling; warnings about gambling from adults in the family; warnings about gambling from, or listening to, peers; having parents who do not gamble; learning about the risks of gambling in school; learning about the risks of gambling from parents; learning about the risks of gambling from peers; adults not involving kids in gambling; and parents/guardians not permitting card games (for money) at home.

*Correlates of problem-gambling severity.* Health and functioning measures were categorized as shown in the tables and included the following variables: grade average; extracurricular activities; lifetime tobacco smoking; lifetime marijuana use; ever and past-30-day alcohol use (categorized as none, light (1–2 drinking-days/month), moderate (3–9 drinking-days/month), and heavy (≤9 days drinking-days/month)); lifetime use of other drugs; caffeine use; past-year sadness or hopelessness for ≤2 weeks; and past-year carrying of a weapon such as a knife, club, or gun to school.

Dichotomous gambling variables (yes/no) were calculated among gamblers and included gambling types (strategic, non-strategic, machine), gambling locations (online, school grounds, casino), gambling triggers (pressure, anxiety), reasons for gambling (excitement, financial, escape, or social), usual company when gambling (family, friends, other adults, strangers, or alone), and weekly time spent gambling (<1 h, ≥ h).

### Data analysis

As in prior analyses (Cavallo et al., 2010; Desai et al., 2010; Grant et al., 2011a, 2011b; Kundu et al., in press; Liu et al., 2011; Potenza et al., 2011; Rahman et al., 2012; Schepis et al., 2008, 2010; Yip et al., 2011), data were double-entered and checked for accuracy. Analyses were conducted using SAS software (Cary, NC). Two-tailed, Pearson chi-square analyses (c^2^) were used to compare characteristics and gambling perceptions of adolescents stratified by fight-involvement status. A Bonferroni correction was applied such that *p*-values of *p* < 0.0025 were considered significant. To produce odds ratios (ORs) and 95% confidence intervals (CIs) as a measure of the magnitude of the association between problem-gambling severity and dependent variables, logistic regression models were constructed for binary outcomes and multinomial logistic regression models for categorical outcomes, stratified according to fight-involvement status. To determine whether fight-involvement status moderated relationships with problem-gambling severity, the entire sample was utilized and main effects for fight involvement and problem-gambling severity, as well as the interaction term (fight-status-by-problem-gambling-severity), were included in the appropriate logistic or multi-nomial logistic regression models. All models were adjusted for gender, grade level, race, Hispanic ethnicity, and family structure (living with one parent, both parents, other). Statistical significance was set at *p* < 0.05.

## Results

Sociodemographic data are displayed in Table 1. Of the 2,276 adolescents studied, 223 (9.8%) indicated past-year serious-fight involvement. One hundred and fifty (69.80%) adolescents who fought were male and 65 (30.23%) were female. One hundred and thirty-four (60.09%) adolescents who fought identified themselves as Caucasian, 32(14.35%) as African-American, 23 (10.31%) as Asian-American, 64 (29.77%) as Hispanic, and 54 (24.22%) as “other race”. Fight involvement was associated with problem-gambling severity (c*^2^* = 93.92; *p* < 0.0001). ARPG was more frequent among adolescents acknowledging serious-fight involvement (hereafter referred to as “fighting adolescents”) than among their non-fighting counterparts(54.7% vs. 24.7%).

**Table 1.** Sociodemographic characteristics stratified by fight-involvement status

Variable	Fight		No fight			
	*N*	%	*N*	%	χ^2^	*p*
Gender					**18.91**	**< .0001**
Male	150	69.80	1104	54.30		
Female	65	30.23	930	45.72		
Race/ethnicity						
Caucasian					**17.88**	**< .0001**
Yes	134	60.09	1508	73.45		
No	89	39.91	545	26.55		
African American					3.04	.081
Yes	32	14.35	216	10.52		
No	191	85.65	1837	89.48		
Asian					**14.99**	**.0001**
Yes	23	10.31	90	4.38		
No	200	89.69	1963	95.62		
Hispanic					**36.93**	**< .0001**
Yes	64	29.77	273	13.96		
No	151	70.23	1683	86.04		
Other race					**11.20**	**.0008**
Yes	54	24.22	318	15.49		
No	169	75.78	1735	84.51		
Grade					6.09	.11
9th	78	35.29	593	28.97		
10th	60	27.15	527	25.74		
11th	54	24.43	561	27.41		
12th	29	13.12	366	17.88		
Living with					**24.51**	**< .0001**
One parent	50	23.26	466	23.04		
2 parents	134	62.33	1441	71.23		
Other	31	14.42	116	5.73		
Gamble 3					**93.92**	< **.0001**
1 (NG)	15	6.73	383	18.66		
2 (LRG)	86	38.57	1163	56.65		
3 (ARPG)	122	54.71	507	24.70		

### Gambling perceptions

Fighting adolescents displayed more permissive views towards gambling on all queried items (all *p* < 0.0012; Table 2). Amongst fighting adolescents, 50.47% to 65.09% viewed specific gambling prevention and other non-permissive measures as important, compared to a range of 65.03% to 89.85% in non-fighting adolescents (Table 2). These measures included items that queried the adolescents on the importance of gambling prevention measures that involved parental oversight of gambling activities, parental non-in-volvement in gambling, and friend/peer non-involvement in gambling. The following items represented the importance of parental oversight: not having access to Internet gambling at home (c*^2^* = 17.75; *p* < 0.0001); parent/guardian strictness about gambling (c*^2^* = 46.14; *p* < 0.001); warnings from adults in family (c*^2^* = 44.91; *p* < 0.0001); learning about the risks of gambling from parents (c*^2^* = 34.66; *p* < 0.0001). The following items represented the importance of parental non-involvement in gambling: having parents who do not gamble (c*^2^* = 30.53; *p* < 0.0001); adults not involving kids in gambling (c*^2^* = 52.41; *p* < 0.0001), and parent/guardian not permitting card games (for money) at home (c*^2^* = 10.48; *p* < 0.0012). The following items represented the importance of friend/peer non-involvement in gambling: hanging out with friends who do not gamble (c*^2^* = 41.83; *p* < 0.0001), warnings from, or listening to, peers (c*^2^* = 54.09; *p* < 0.0001); learning about the risks of gambling from peers (c^2^ = 26.73; *p* < 0.0001). In addition, fighting adolescents indicated significantly greater parental approval of gambling (c^2^ = 72.20, *p* < 0.0001), as well as greater concern about the gambling of a close family member (c^2^ = 23.18; *p* < 0.0001).

**Table 2.** Gambling perceptions in fighting and non-fighting adolescents

Variable/Category	Fight [JV(%)]	No Fight [JV(%)]	χ^2^ statistics	
			χ^2^	*p* value
Parent perception about gambling			**72.20**	**<.0001**
Disapprove	66 (37.50)	740 (42.38)		
Neither approve nor disapprove	61 (34.66)	866 (49.60)		
Approve	49 (7.84)	140 (8.02)		
Importance for preventing gambling problems in teens				
Checking identification for purchasing lottery tickets			**58.64**	**< .0001**
Important	129 (61.14)	1605 (82.99)		
Not important	82 (38.86)	329(17.01)		
Hanging out with friends who do not gamble			**41.83**	**< .0001**
Important	113 (53.55)	1427 (74.52)		
Not important	98 (46.45)	488 (25.48)		
Participating in activities that are fun and free of gambling			**59.99**	**< .0001**
Important	126 (60.58)	1589 (82.89)		
Not important	82 (39.42)	328 (17.11)		
Fear of losing valuable possessions, close friends, and relatives			**106.01**	**< .0001**
Important	138 (65.09)	1717 (89.85)		
Not important	74 (34.91)	194 (10.15)		
Advertisements that show the problems associated with gambling			**37.26**	**< .0001**
Important	121 (57.89)	1465 (77.11)		
Not important	8842.11	435 (22.89)		
Not having access to Internet gambling at home			**17.75**	**< .0001**
Important	107 (50.47)	1239 (65.14)		
Not important	105 (49.53)	663 (34.86)		
Parent/Guardian strictness about gambling			**46.14**	**< .0001**
Important	128 (60.66)	1537 (80.81)		
Not important	83 (39.34)	365 (19.19)		
Warnings from adults in family			**44.91**	**< .0001**
Important	125 (60.68)	1537 (80.77)		
Not important	81 (39.32)	366 (19.23)		
Warnings from, or listening to, peers			**54.09**	**< .0001**
Important	126 (60.29)	1551 (81.85)		
Not important	83 (39.71)	344 (18.15)		
Having parents who do not gamble			**30.53**	**< .0001**
Important	132 (62.56)	1507 (79.27)		
Not important	79 (37.44)	394 (20.73)		
Learning about the risks of gambling in school			**30.04**	**< .0001**
Important	125 (59.24)	1453 (76.51)		
Not important	86 (40.76)	446 (23.49)		
Learning about the risks of gambling from parents			**34.66**	**< .0001**
Important	135 (64.29)	1549 (81.48)		
Not important	75 (35.71)	352 (18.52)		
Learning about the risks of gambling from peers			**26.73**	**< .0001**
Important	132 (62.26)	1486 (78.13)		
Not important	80 (37.74)	416(21.87)		
Adults not involving kids in gambling			**52.41**	**< .0001**
Important	130 (61.90)	1569 (82.71)		
Not important	80 (38.10)	328 (17.29)		
Parent/Guardian not permitting card games (for money) at home			**10.48**	**.0012**
Important	114(53.77)	1235 (65.03)		
Not important	98 (46.23)	664 (34.97)		
Family concern			**23.18**	**< .0001**
Yes	48 (23.53)	221 (11.67)		
No	156 (76.47)	1673 (88.33)		

### Health/functioning measures

Health and functioning data are displayed in Table 3 and Supplemental Table 1. Among fighting adolescents, both LRG and ARPG groups were more likely than non-gamblers to report occasional smoking (OR = 7.24, 95% CI =[1.27–41.32], OR = 16.02, 95% CI = [2.58–99.53]), regular smoking (OR = 7.19, 95% CI = [1.22–42.54], OR = 24.54, 95% CI = [3.83–157.40]), and lifetime alcohol consumption (OR = 9.51, 95% CI = [2.96–128.57]; OR = 7.45, 95% CI =[1.35–41.11]).

**Table 3.** Health and well-being measures and problem-gambling severity in fighting and non-fighting adolescents

Variable	Fight		No fight		Interaction OR (Fight vs. No fight)	
	LRG vs. NG	ARPG vs. NG	LRG vs. NG	ARPG vs. NG	LRG vs. NG	ARPG vs. NG
	OR (95% CI)	OR (95%CI)	OR (95% CI)	OR (95% CI)	OR (95% CI)	OR (95% CI)
Any extracurricular	0.24 (0.03-2.07)	0.17(0.02-1.44)	**1.59 (1.22-2.09)**	**2.01 (1.43-2.81)**	0.17(0.02-1.41)	0.12(0.02-1.01)
activities						
Grade average						
A's and B's	Ref.	Ref.	Ref.	Ref.	Ref.	Ref.
Mostly C's	0.83 (0.22-3.19)	1.16(0.29-4.63)	1.30 (0.98-1.74)	**1.51 (1.07-2.11)**	0.77 (0.21-2.86)	0.82 (0.22-3.04)
D's or lower	3.16 (0.33-30.61)	9.12(0.93-89.19)	0.88 (0.58-1.33)	1.21 (0.76-1.93)	3.50 (0.36-33.72)	4.81 (0.51-45.47)
Substance use						
*Marijuana use, lifetime*	0.91 (0.23-3.61)	1.23 (0.30-5.00)	**1.76 (1.33-2.34)**	**2.71 (1.95-3.76)**	0.76(0.19-3.02)	0.82 (0.21-3.26)
*Other drug use, lifetime*	1.36 (0.35-5.24)	3.19(0.82-12.46)	**1.92 (1.04-3.56)**	**3.20 (1.64-6.22)**	0.63 (0.15-2.69)	0.94 (0.22-3.93)
*Smoking, lifetime*						
Never	Ref.	Ref.	Ref.	Ref.	Ref.	Ref.
Occasionally	**7.24 (1.27-41.32)**	**16.02 (2.58-99.53)**	**1.83 (1.33-2.52)**	**2.82 (1.95-4.08)**	3.07 (0.57-16.70)	4.32 (0.78-23.94)
Regularly	**7.19 (1.22-12.54)**	**24.54 (3.83-157.40;**	**) 1.84 (1.20-2.81)**	**2.76 (1.69-4.49)**	3.11 (0.54-17.83)	**7.59 (1.30-44.23)**
Alcohol use						
*Alcohol use, lifetime*	**19.51 (2.96-128.57**	**) 7.45 (1.35-41.11)**	**3.78 (2.67-5.35)**	**4.71 (3.01-7.36)**	3.41 (0.62-18.72)	1.42 (0.31-6.40)
*Alcohol use, current*						
Never regular	Ref.	Ref.	Ref.	Ref.	Ref.	Ref.
Light	3.14(0.11-91.74)	8.78 (0.27-288.74	1.31 (0.83-2.05)	1.65 (0.97-2.79)	1.24 (0.06-24.60)	1.46 (0.08-28.22)
Moderate	2.81 (0.14-57.20)	2.61 (0.11-62.50)	**1.77 (1.10-2.85)**	**2.68 (1.55-4.63)**	1.12(0.08-15.34)	0.59 (0.04-8.20)
Heavy	−	−	**2.56 (1.19-5.50)**	**5.37 (2.34-12.30)**	−	−
Caffeine use						
None	Ref.	Ref.	Ref.	Ref.	Ref.	Ref.
1-2 per day	3.51 (0.54-22.63)	3.19(0.47-21.82)	**1.60 (1.12-2.16)**	1.23 (0.86-1.77)	1.43 (0.24-8.37)	1.53 (0.26-8.91)
3+ per day	2.20 (0.41-11.79)	3.91 (0.70-21.90)	**2.88 (1.95-1.25)**	**3.10 (1.97-4.83)**	0.44 (0.09-2.15)	0.59 (0.12-2.85)
Mood						
Dysphoria/						
Depression	0.55 (0.14-2.13)	0.78 (0.20-3.04)	1.22 (0.88-1.68)	**2.16 (1.47-3.17)**	0.48 (0.13-1.77)	0.37 (0.10-1.34)
Aggression						
Carry weapon	**4.15 (1.05-16.39)**	**16.50 (3.85-70.69)**	**1.94 (1.29-2.92)**	**3.21 (2.09-4.95)**	1.59(0.39-6.43)	3.16(0.76-13.18)
Weight						
Normal	Ref.	Ref.	Ref.	Ref.	Ref.	Ref.
Underweight	1.16(0.10-13.50)	2.00 (0.16-24.84)	1.09 (0.70-1.69)	1.04(0.60-1.80)	1.59(0.16-15.72)	1.57 (0.16-15.57)
Overweight	1.35 (0.26-6.88)	0.66 (0.12-3.50)	0.77 (0.55-1.10)	0.90 (0.60-1.36)	1.41 (0.32-6.28)	0.73 (0.16-3.36)
Obese	−	−	0.97 (0.56-1.70)	1.40(0.75-2.62)	−	−

**Supplemental Table 1.** Health and well-being measures and problem-gambling severity in fighting and non-fighting adolescents: Chi-square analyses

Variable	Fight								No fight							
	NG		LRG		ARPG		χ^2^	*p*	NG		LRG		ARPG		χ^2^	*p*
	*N*	%	*N*	%	*N*	%			*N*	%	*N*	%	*N*	%		
Any extracurricular activities	13	86.67	64	74.42	93	76.23	1.06	.59	259	67.62	887	76.27	399	78.70	**15.85**	**.0004**
Grade average							5.44	0.25							**20.24**	**.0004**
A's and B's	8	53.33	35	41.67	38	32.20			236	63.27	656	57.75	244	49.80		
Mostly C's	5	33.33	30	35.71	41	34.75			93	24.9	361	31.78	173	35.31		
D's or lower	2	13.33	19	22.62	39	33.05			44	11.80	119	10.48	73	14.90		
Substance use																
*Smoking, lifetime*							**14.77**	**0.0052**							**34.67**	< **.0001**
Never	9	64.29	25	30.12	22	19.30			269	73.10	696	60.95	265	53.54		
Occasionally	2	14.29	26	31.33	34	29.82			63	17.12	287	25.13	153	30.91		
Regularly	3	21.43	32	38.55	58	50.88			36	9.78	159	13.92	77	15.56		
*Marijuana, lifetime*	9	64.29	58	72.50	88	75.86	0.98	.61	99	28.05	443	40.38	237	50.11	**40.84**	< **.0001**
*Alcohol, sip*	9	64.29	78	95.12	102	87.93	**12.15**	**.0023**	262	73.60	988	89.98	432	90.19	**69.56**	< **.0001**
*Alcohol, current*							3.32	0.77							**20.55**	**0.0022**
Never regular	1	20.00	9	17.65	8	14.29			63	41.18	239	30.06	91	25.07		
Light	1	20.00	9	17.65	17	30.36			44	28.76	227	28.55	98	27.00		
Moderate	2	40.00	18	35.29	14	25.00			35	22.88	239	30.06	116	31.96		
Heavy	1	20.00	15	29.41	17	30.36			11	7.19	90	11.32	58	15.98		
*Other drug, lifetime*	5	33.33	26	37.68	54	53.47	5.16	0.076	15	4.79	76	8.24	51	13.01	**15.38**	**.0005**
Caffeine use							4.07	0.40							**56.49**	< **.0001**
None	3	21.43	15	18.52	18	14.88			119	32.16	212	18.53	109	21.80		
1-2 per day	3	21.43	32	39.51	37	50.58			198	53.51	620	54.20	229	45.80		
3+ per day	8	57.14	34	41.98	66	54.55			53	14.32	312	27.27	162	32.40		
Mood							1.632	0.44								
Dysphoria/depression	10	66.67	38	48.72	58	52.25			68	18.38	216	19.30	110	22.59	2.99	.22
Aggression							**27.86**	**< .0001**							**77.50**	< **.0001**
Carry weapon	5	33.33	47	56.63	100	84.03			37	9.74	223	19.26	168	33.33		
Weight							1.93	0.93							7.48	.28
Normal	9	64.29	45	57.69	56	62.22			224	66.08	731	68.70	295	64.69		
Underweight	2	14.29	9	11.54	11	12.22			35	10.32	107	10.06	38	8.33		
Overweight	3	21.43	18	23.08	16	17.78			60	17.70	161	15.13	85	18.64		
Obese	0	0	6	7.69	7	7.78			20	5.90	65	6.11	38	8.33		

Among non-fighting adolescents, both LRG and ARPG groups were more likely than non-gamblers to report occasional smoking (OR = 1.83, 95% CI = [1.33–2.52]; OR =2.82, 95% CI = [1.95–4.08]) and regular smoking (OR =1.84, 95% CI = [1.20–2.81]; OR = 2.76, 95% CI =[1.69–4.49]). LRG and ARPG groups were also more likely than non-gamblers to report ever having consumed alcohol (OR = 3.78, 95% CI = [2.67–5.35]; OR = 4.71, 95% CI =[3.01–7.36]), using marijuana (OR = 1.76, 95% CI =[1.33–2.34]; OR = 2.71, 95% CI = [1.95–3.76]), current moderate alcohol use (OR = 1.77, 95% CI = [1.10–2.85]; OR = 2.68, 95% CI = [1.55–4.63]), current heavy alcohol use (OR = 2.56, 95% CI = [1.19–5.50]; OR = 5.37, 95% CI =[2.34–12.30]), or other drug use (OR = 1.92, 95% CI =[1.04–3.56]; OR = 3.20, 95% CI = [1.64–6.22]). Among fighting adolescents, LRG and ARPG groups were more likely than non-gamblers to report past-month weapon possession (OR = 4.15, 95% CI = [1.05–16.39]; OR = 16.50, 95% CI = [3.85–70.69]). Among non-fighting adolescents, LRG and ARPG groups were more likely than non-gamblers to report past-month weapon possession (OR = 1.94, 95% CI = [1.29–2.92]; OR = 3.21, 95% CI = [2.09–4.95]). Among non-fighting adolescents, ARPG adolescents were more likely than non-gamblers to report a grade average of mostly C's (OR = 1.51, 95% CI = [1.07–2.11]) as well as dysphoria/depression (OR = 2.16, 95% CI =[1.47–3.17]).

Interaction analyses revealed a stronger relationship between ARPG and regular smoking in the fighting versus non-fighting groups (OR = 7.59; 95% CI = [1.30–44.23]). This means that the association between ARPG and smoking was over seven times stronger in fighting adolescents than in non-fighting adolescents.

### Gambling characteristics

Gambling characteristics are displayed in Table 4 and Supplemental Table 2. Among fighting adolescents, ARPG adolescents were more likely than LRG adolescents to gamble online, in school, and at the casino (OR = 3.67, 95% CI =[1.82–7.39]; OR = 2.85, 95% CI = [1.42–5.72]; OR = 4.51, 95% CI = [2.05–9.93]); experience pressure and anxiety as triggers to gamble (OR = 9.89, 95% CI = [3.11–31.41]; OR = 19.92, 95% CI = [5.09–77.94]); gamble for financial reasons, escape, or social reasons (OR = 2.26, 95% CI =[1.14–4.50], OR = 2.73, 95% CI = [1.42–5.25]; OR = 2.53, 95% CI = [1.30–4.93]); and gamble with strangers or alone (OR = 3.48, 95% CI = [1.43–8.48]; OR = 3.17, 95% CI =[1.24–8.13]).

**Table 4.** Gambling measures and problem-gambling severity in fighting and non-fighting adolescents

Variable	Fight	No fight	Interaction OR (Fight vs. No fight)
	ARPG vs. LRG	ARPG vs. LRG	ARPG vs. LRG
	OR (95% CI)	OR (95% CI)	OR (95% CI)
Gambling type			
Strategic	4.97 (0.59-41.57)	**5.10 (1.98-13.13)**	0.55 (0.08-3.91)
Non-strategic	1.22 (0.55-2.68)	**1.68 (1.29-2.20)**	0.76 (0.35-1.65)
Machine	1.63 (0.77-3.43)	**2.20 (1.74-2.79)**	0.59(0.29-1.22)
Gambling location			
Online	**3.67 (1.82-7.39)**	**2.55 (1.92-3.39)**	1.16(0.57-2.39)
School gambling	**2.85 (1.42-5.72)**	**4.28 (3.32-5.52)**	0.592 (0.29-1.23)
Casino	**4.51 (2.05-9.93)**	**3.09 (2.02-4.72)**	1.44 (0.61-3.38)
Triggers for gambling			
Pressure	**9.89 (3.11-31.41)**	**3.11 (2.05-4.73)**	2.59 (0.80-8.34)
Anxiety	**19.92 (5.09-77.94)**	**13.30 (5.73-30.85)**	1.08(0.24-4.83)
Reasons why gamble			
Excitement	2.05 (0.98-4.30)	**3.10 (2.33-4.15)**	0.63 (0.29-1.33)
Financial reasons	**2.26 (1.14-4.50)**	**3.48 (2.71-4.48)**	0.69 (0.34-1.37)
Escape	**2.73 (1.42-5.25)**	**2.46 (1.93-3.14)**	1.02 (0.53-1.97)
Social reasons	**2.53 (1.30-4.93)**	**1.78 (1.40-2.25)**	1.28(0.66-2.47)
People gamble with			
Family	0.93 (0.50-1.74)	**1.65 (1.31-2.08)**	0.62 (0.33-1.17)
Friends	0.61 (0.29-1.28)	**2.16 (1.61-2.90)**	0.28(0.14-0.59)
Other adults	1.26(0.66-2.42)	**2.41 (1.86-3.12)**	0.50 (0.26-0.97)
Strangers	**3.48 (1.43-8.48)**	**4.66 (2.94-7.38)**	0.82 (0.31-2.17)
Alone	**3.17 (1.24-8.13)**	**3.53 (2.30-5.42)**	1.22 (0.45-3.28)
Time spent gambling			
1 hour or less	Ref.		
2+ hours/week	**5.68 (2.46-13.12)**	**4.47 (3.13-6.37)**	0.89 (0.38-2.07)
Age of onset of gambling			
≤ 8 years old	Ref.	Ref.	Ref.
9-11 years old	1.39(0.51-3.80)	1.12(0.70-1.77)	1.17(0.41-3.28)
12-14 years old	0.57(0.24-1.35)	0.88 (0.59-1.31)	0.64 (0.26-1.58)
≥ 15 years old	0.34(0.12-0.91)	0.68(0.45-1.04)	0.51 (0.19-1.42)

LRG = low-risk gambling; ARPG = at-risk/problem gambling.

**Supplemental Table 2.** Gambling measures and problem-gambling severity in fighting and non-fighting adolescents: Chi-square analyses

Variable	Fight						No fight					
	LRG		ARPG		χ^2^	*p*	LRG		ARPG		χ^2^	*p*
	*N*	%	*N*	%			*N*	%	*N*	%		
Gambling type												
Strategic	82	95.35	120	98.36	1.63	0.20	1097	94.3	501	98.8	**17.27**	**< .0001**
Non-strategic	66	76.74	97	79.51	0.23	0.63	787	67.67	375	73.96	**6.61**	**0.010**
Machine	59	68.60	95	77.87	2.25	0.13	422	36.29	312	61.54	**91.40**	**< .0001**
Gambling location												
Online	22	25.58	64	53.78	**16.30**	**< .0001**	138	11.98	150	29.94	**78.29**	**< .0001**
School gambling	44	51.76	90	74.38	**11.23**	**0.0008**	286	24.70	319	63.42	**227.06**	**< .0001**
Casino	14	16.28	53	43.80	**17.40**	**< .0001**	53	4.59	66	13.20	**38.71**	**< .0001**
Triggers for gambling												
Pressure	4	4.76	40	33.90	**24.45**	**< .0001**	47	4.06	70	13.94	**52.24**	**< .0001**
Anxiety	4	4.76	45	39.13	**30.89**	**< .0001**	9	0.86	47	9.81	**74.59**	**< .0001**
Reasons why gamble												
Excitement	59	68.60	101	82.79	**5.72**	**0.0168**	700	60.19	423	83.43	**86.60**	**< .0001**
Financial reasons	49	56.98	91	74.59	**7.11**	**0.0077**	495	42.56	380	74.95	**148.49**	**< .0001**
Escape	28	32.56	71	58.20	**13.29**	**0.0003**	252	21.67	211	41.62	**70.12**	**< .0001**
Social reasons	34	39.53	77	63.11	**11.27**	**0.0008**	373	32.07	247	48.72	**41.91**	**< .0001**
People gamble with												
Family	42	48.84	68	55.74	0.96	0.33	479	41.19	271	53.45	**21.47**	**< .0001**
Friends	63	73.26	86	70.49	0.19	0.66	776	66.72	425	83.83	**51.13**	**< .0001**
Other adults	28	32.56	52	42.62	2.16	0.14	202	17.37	173	34.12	**56.91**	**< .0001**
Strangers	9	10.47	43	35.25	**16.52**	**< .0001**	33	2.84	77	15.19	**87.52**	**< .0001**
Alone	7	8.14	37	30.33	**14.89**	**0.0001**	47	4.04	70	13.81	**51.68**	**< .0001**
Time spent gambling					**17.28**	**< .0001**					**123.81**	**< .0001**
1 hour or less	58	79.45	57	49.14			890	93.19	332	71.40		
2+ hours/week	15	20.55	59	50.86			65	6.81	133	28.60		
Age of onset of gambling					**13.55**	**0.0036**					6.20	.10
< 8 years old	21	28.38	47	41.23			94	10.99	64	13.45		
9-11 years old	10	13.51	30	26.32			129	15.09	83	17.44		
12-14 years old	23	31.08	24	21.05			330	38.60	190	36.54		
≥ 15 years old	20	27.03	13	11.40			302	35.32	139	29.20		

Among non-fighting adolescents, ARPG adolescents were more likely than LRG adolescents to engage in strategic, non-strategic and machine gambling (OR = 5.10, 95% CI = [1.98–13.13]; OR = 1.68, 95% CI = [1.29–2.20]; OR =2.20, 95% CI = [1.74–2.79]); gamble online, in school, and at the casino (OR = 2.55, 95% CI = [1.92–3.39]; OR = 4.28, 95% CI = [3.32–5.52]; OR = 3.09, 95% CI = [2.02–4.72]); experience pressure and anxiety as triggers (OR = 3.11, 95% CI = [2.05–4.73]; OR = 13.30, 95% CI = [5.73–30.85]); gamble for financial reasons, social reasons, and excitement (OR = 3.48, 95% CI = [2.71–4.48]; OR = 1.78, 95% CI =[1.40–2.25]; OR = 3.10, 95% CI = [2.33–4.15]); and gamble with family, friends, other adults, strangers, or alone (OR = 1.65, 95% CI = [1.31–2.08]; OR = 2.16, 95% CI =[1.61–2.90]; OR = 2.41, 95% CI = [1.86–3.12]; OR =4.66, 95% CI = [2.94–7.38]; OR = 3.53, 95% CI =[2.30–5.42]).

Interaction analyses did not identify any significant effects, suggesting that the gambling-related correlates of problem-gambling severity were similar across fighting and non-fighting groups.

## Discussion

To our knowledge, this is the first study to investigate differences in gambling perceptions, attitudes and behaviors and problem-gambling-severity correlates in adolescents stratified by past-year involvement in serious physical fights that required medical attention. Consistent with our first hypothesis, fight-involvement status was associated with problem-gambling severity, with a greater proportion of ARPG amongst fighting versus non-fighting adolescents. Consistent with our second hypothesis, fighting versus non-fighting adolescents reported more permissive attitudes toward gambling. Our third and fourth hypotheses were largely not supported as the relationship between problem gambling severity and health and gambling characteristics appeared similar across the two groups of adolescents, with the exception of regular smoking. Clinical implications are described below.

### Problem-gambling severity

Associations between violent behaviors and problem-gambling severity have been reported previously (Afifi et al., 2010; Brasfield et al., 2012; Chaumeton, 2011; Goldstein et al., 2009; Korman et al., 2008; Potenza et al., 2011; Proimos et al., 1998). Our finding of an association between ARPG and serious physical fights suggests that youth engaging in fights may be at risk for gambling problems; alternatively youth engaging in gambling may potentially be at risk for fighting. As physical fighting among adolescents is commonly visible to adults who may witness the act or observe the injuries, it should help provide insight into less observable risk behaviors, such as gambling, in which the adolescent may be involved. Schools might consider targeting aggressive behaviors with educational interventions; e.g., teaching individuals detained for physical fighting about the potential risks of gambling. School policies could also consider educating parents of adolescents who fight about the relationship between fighting and gambling in adolescents.

Although reasons behind an association between violent behaviors and gambling are undetermined, several possibilities include motivational factors (e.g., gambling as a method of escape from distressing situations) or shared behavioral tendencies (diminished self-control; Boughton & Falenchuk, 2007; Crisp et al., 2004; Ledgerwood & Petry, 2006; Li, 2007; Walker, Hinch & Weighill, 2005). Poor impulse control should be further investigated in fighting adolescents, which could link to both perpetrators and victims of physical violence. The extent to which adolescents involved in fights gamble to escape should also be further studied, particularly amongst youth who are bullied, as our data indicate a greater percentage of fighting adolescents reporting gambling to escape (48%) than non-fighting adolescents (27%). This finding indicating more frequent motivations relating to gambling to escape amongst fighting adolescents reporting resulting injuries may suggest a greater likelihood of losing the fight or being the victims of bullying. Additional research is needed to investigate directly this possibility, and if this hypothesis is upheld, it may be particularly useful to assess gambling behaviors in adolescent victims of bullying.

### Gambling attitudes and perceptions

Fighting versus non-fighting adolescents reported more permissive views toward gambling across a broad range of measures. More adolescents in the fight (versus non-fight) group indicated that their parents approved of gambling, possibly suggesting a permissive parenting style amongst these parents. Fighting versus non-fighting adolescents rated the multiple gambling-related efforts involving parental oversight as less important including parent/guardian strictness about gambling, warnings from adults in family, learning about the risks of gambling from parents, and not having access to Internet gambling at home. Permissive parenting, characterized by a lack of parental monitoring (Ginsburg, Durbin, Garcia-Espana, Kalicka & Winston, 2009), is linked to internalizing and externalizing behavioral problems in youth (Alizadeh, Talib, Abdullah & Mansor, 2011), and may represent an underlying factor in adolescents who engage in physical fighting and gambling. Low parental monitoring has been linked to both gambling and physical fighting in adolescents while high levels protect against these risk behaviors (Curtner-Smith & MacKinnonLewis, 1994; Magoon & Ingersoll, 2006; Rudatsikira, Mataya, Siziya & Muula, 2008).

Fighting adolescents also rated measures reflecting parental involvement in gambling, such as having parents who do not gamble and parents who do not involve their children in gambling activities, as significantly less important than adolescents who did not fight. Parents may directly influence their child's engagement in risk behaviors by openly participating in these activities themselves and/or involving their child in such behaviors. Children of parents who gamble and of parents who endorse violent behaviors have been shown to engage in similar behaviors (Farrell, Henry, Mays & Schoeny, 2011; Lesieur & Klein, 1987; Ohene, Ireland, McNeely & Borowsky, 2006; Winters, Bengston, Dorr & Stinchfield, 1998; Winters, Stinchfield & Fulkerson, 1993). These associations should be considered in the development of school disciplinary policies for adolescents who fight, as a greater emphasis on the parent-child relationship may be helpful in reducing other risk behaviors in these adolescents. For example, school administrators or teachers could educate parents of fighting adolescents on the protective role of parental monitoring and the potential risks of openly engaging in gambling or violent behaviors.

In addition, fighting versus non-fighting adolescents rated as less important those measures reflecting friend/peer involvement in gambling, such as hanging out with friends who do not gamble, warnings from or listening to peers about gambling, and learning about the risks of gambling from peers. Such responses suggest a greater propensity for fighting youth to consider gambling less risky or problematic, including with respect to peer advice about gambling-related risks. Data indicate strong links between delinquent peer associations and problem behaviors that include gambling and physical aggression (Brown & Wolfe, 1994; Farrell et al., 2011; Hardoon & Derevensky, 2001; Kearney & Drabman, 1992). Developing approaches to change gambling attitudes amongst fighting youth, including with respect to peer involvement, represents an important effort.

Fighting adolescents more frequently acknowledged concerns about a family member's gambling, suggesting that adolescents who witness parents with poor control over their gambling may be more likely to get into fights at school. The extent to which this relationship underlies the observed findings, as well as the extent to which other related factors (e.g., stress or trauma exposure, each of which has been linked to gambling and violence [Bergevin, Gupta, Derevensky & Kaufman, 2006; Kaplan, Madden, Mijanovich & Purcaro, 2012; Kausch, Rugle & Rowland, 2006; Schiff et al., 2012]) might mediate such a relationship, warrants additional investigation. Additionally, the extent to which individual differences relating to impulse control might mediate relationships between stress and gambling (as has been observed between stress and hazardous drinking in adults [Hamilton, Ansell, Reynolds, Potenza & Sinha, 2013]) warrants further investigation. Such information could inform the development and implementation of interventions (e.g., mindfulness-based stress reduction) to prevent youth violence and gambling problems (de Lisle, Dowling & Allen, 2011; Robins, Keng, Ekblad & Brantley, 2012).

### Correlates of problem-gambling severity

With the exception of regular smoking, the correlates of problem-gambling severity with measures of functioning and gambling characteristics were largely similar amongst fighting and non-fighting adolescents. The stronger association between problem-gambling severity and tobacco smoking amongst fighting adolescents appears consistent with reported associations between gambling, violent behaviors, and alcohol and substance use (Brasfield et al., 2012; Wanner, Vitaro, Charbonneau & Tremblay, 2009). Although the nature of these associations is undetermined, similarities in personal dispositions, familial qualities, and peer influences have been shown in adolescents who engage in gambling, compulsive substance and alcohol use, and delinquent behaviors including physical violence; these factors include impulsivity, poor parental supervision, and deviant peers (Wanner et al., 2009). Adolescents may also be especially likely to engage in high-risk behaviors such as cigarette smoking, physical fighting and gambling for social reasons such as peer pressure and improving popularity (Brady, Song & Halpern-Felsher, 2008; Johnson, Frattaroli, Wright, Pearson-Fields & Cheng, 2004; Langhinrichsen-Rohling, Rohde, Seeley & Rohling, 2004).

### Strengths and limitations

This study has multiple strengths including a large sample size that is similar in composition to Connecticut census data, as previously described (Cavallo et al., 2010; Desai et al., 2010; Grant et al., 2011a, 2011b; Kundu et al., in press; Liu et al., 2011; Potenza et al., 2011; Rahman et al., 2012; Schepis et al., 2008, 2010; Yip et al., 2011). Limitations also exist. First, the number of adolescents involved in serious physical fights was relatively small, limiting the power to detect interaction effects. Second, although the question determining fight-involvement status is derived from the widely used Youth Child Risk Behavior Survey (thus facilitating comparisons across studies), it does not differentiate between levels of violence, such as the amount or extent of physical fights, or whether these adolescents were perpetrators or victims of physical violence. Future research should investigate these areas with respect to problem-gambling severity. Such information might be best obtained with the input of involved school officials as adolescents involved in fights may be unwilling to admit to either perpetration or victimization, or may be inaccurate in their assessments thereof. Third, as the sample is from Connecticut, it is not nationally representative and findings may not generalize. Fourth, the study was cross-sectional, limiting the ability to fully examine the nature of the observed associations. For example, it cannot be determined whether fighting in adolescence leads to gambling or gambling leads to fighting behaviors; thus, longitudinal studies are needed. Fifth, multiple measures, including assessments of depressive and aggressive features, used non-diagnostic and dichotomous measurements. Future studies using more clinically valid measurements may be valuable to better understand relationships between problem-gambling severity and health/ functioning measures.

## Conclusions

The current study demonstrates that adolescents involved in serious physical fights are more likely to report more permissive gambling-related perceptions and attitudes, exhibit more risky/problematic gambling and demonstrate stronger associations between tobacco smoking and problem-gambling severity than adolescents who do not physically fight. Such findings highlight the need for more research into the etiologies of these relationships. Improved educational prevention and interventional efforts for adolescents who fight that also incorporate teachings on more discrete risk behaviors such as gambling, may be useful in targeting commonalities of both risk behaviors.
